# The Relationship Between Salivary Redox, Diet, and Food Flavor Perception

**DOI:** 10.3389/fnut.2020.612735

**Published:** 2021-01-28

**Authors:** Mathieu Schwartz, Fabrice Neiers, Gilles Feron, Francis Canon

**Affiliations:** Centre des Sciences du Goût et de l'Alimentation, UMR1324 INRA, UMR6265 CNRS Université de Bourgogne, Dijon, France

**Keywords:** saliva, antioxidant capacity, diet, flavor, redox, perception, antioxidant, salivary proteins

## Abstract

The mouth is the gateway for entrance of food and microorganisms into the organism. The oral cavity is bathed by saliva, which is thus the first fluid that food and microorganisms will face after their entrance. As a result, saliva plays different functions, including lubrication, predigestion, protection, detoxification, and even transport of taste compounds to chemoreceptors located in the taste buds. To ensure its function of protection, saliva contains reactive harmful compounds such as reactive oxygen species that are controlled and neutralized by the antioxidant activity of saliva. Several antioxidant molecules control the production of molecules such as reactive oxygen compounds, neutralize them and/or repair the damage they have caused. Therefore, a balance between reactive oxidant species and antioxidant compounds exists. At the same time, food can also contain antioxidant compounds, which can participate in the equilibrium of this balance. Numerous studies have investigated the effects of different food components on the antioxidant capacity of saliva that correspond to the ability of saliva to neutralize reactive oxygen species. Contradictory results have sometimes been obtained. Moreover, some antioxidant compounds are also cofactors of enzymatic reactions that affect flavor compounds. Recent studies have considered the salivary antioxidant capacity to explain the release of flavor compounds *ex vivo* or *in vivo*. This article aims to review the effect of food on the antioxidant capacity of saliva and the impact of salivary antioxidant capacity on flavor perception after a brief presentation of the different molecules involved.

## Introduction

This review reports the relationships that have been established in the literature between the salivary antioxidant capacity, the diet and the perception of food flavor. As several reviews have already approached the relationship between salivary antioxidant capacity and pathologies (1, 2), this aspect will only be briefly discussed in this review.

Saliva is a complex biological fluid that plays an important role in bodily protection and consequently in health (3). In addition to protecting the oral cavity against microorganisms and abrasion by food particles (4, 5), saliva ensures several functions in food perception (6). Indeed, saliva ensures the transport of tastants and trophic factors to the taste buds (7), and thus allows the detection of food compounds including energetic and toxic compounds by the taste receptors. Protection of the oral cavity involves the secretion of numerous salivary proteins such as immunoglobulins or enzymes that regulate the production of reactive oxygen species (ROS) and reactive nitrogen species (RNS). ROS and RNS react with biomolecules including proteins, lipids and nucleic acids. Consequently, these species are toxic toward epithelial cells and the microorganisms living on the oral tissues. Thus, the regulation of these reactive species through the production of antioxidant compounds is essential for the organism. Food also contains oxidizing, reducing and antioxidizing compounds that can affect, at least temporally, the antioxidant status of the oral cavity. Moreover, the antioxidant capacity of saliva has recently been suggested to modulate the metabolization of flavor compounds (8, 9) and thus impact their release (8–11) and effect on their perception (12). The first part of this review introduces the salivary redox status and the involved species, the second section reports on the relationship between salivary antioxidant capacity and physiologic status capacity and finally we present the links between salivary redox, diet and food perception.

## Salivary Redox Status

### Reactive Oxygen Species

#### Reactive Oxygen Species Origins

Molecular dioxygen is essential for cellular respiration but is toxic. Even if this toxicity is low, it can lead to the formation of much more reactive species and consequently toxic species called reactive oxygen species (ROS). ROS can be generated by many physiological processes such as cellular respiration but is also used as a defense body during an immune response. ROS include oxygen radical forms that are very reactive despite a very short lifespan (less than a millisecond). The effects of these radical forms mainly result from their reactions with lipids, nucleic acids (13) and proteins (14). In the mouth, ROS are generated in the oral epithelium and directly in the saliva. ROS production in the oral cavity regulates the oral microbiota. Furthermore, ROS production is also physiologically limited to prevent many pathologies including inflammatory syndromes or even oral cancers such as oral leukoplakia (15). Many exogenous factors can lead to the deregulation of the redox balance by acting directly on the oral cavity, namely, in a non-exhaustive manner: the use of tobacco (16), certain pharmaceutical molecules, and many pro- or antioxidants present in the food naturally or artificially as certain additives. Interestingly, the deregulation of saliva redox balance is a good diagnostic indicator for many pathologies that are not only directly linked to the oral cavity (HIV, diabetes, renal dysfunction, etc.) (1).

#### Reactive Oxygen Species Types

There are many types of ROS. Without being exhaustive, those that will be important from the point of view of their consequences in terms of reactivity with biological molecules or generation of other ROS will be mentioned here.

Superoxide anion (${\text{O}}_{2}^{\cdot -}$) is the precursor of many ROS. The superoxide anion is produced by the monoelectronic reduction of dioxygen naturally during cellular respiration but can also result from enzymatic production [e.g., xanthine oxidase in milk (17)]. Superoxide anion reacts with many endogenous (e.g., haemoproteins) or exogenous molecules, especially those contained in food (e.g., sulfites, thiols, and quinones) (18).

Hydrogen peroxide (H_2_O_2_) results from the dismutation of the superoxide anion. This dismutation can be catalyzed by superoxide dismutase, an enzyme found in human saliva. Hydrogen peroxide can also be produced and released in saliva by oral microbiota (19). Importantly, hydrogen peroxide can cross cell membranes and serve as a secondary messenger in many cellular processes (20). The diffusion capacity of hydrogen peroxide has a high impact because it is the source of one of the most reactive ROS: the hydroxyl radical (OH·). Superoxide anion also leads to the generation of the hydroxyl radical. This highly reactive ROS can react with many molecules although its high reactivity limits its diffusion. Two main reactions lead to its production: the Haber-Weiss reaction between superoxide anion and hydrogen peroxide (21) and the Fenton reaction between reduced iron (Fe^2+^) and hydrogen peroxide (22).

Other ROS can be synthesized enzymatically, such as nitric oxide (∙NO) by nitrite oxide synthase, which is produced in submaxillary glands (23), from arginine (24) or hypochlorous acid from chloride and hydrogen peroxide by myeloperoxidase, which is also present in saliva (see paragraph Elimination of ROS). Hydrogen peroxide is particularly toxic in its ability to form the hydroxyl radical by reacting directly with superoxide anion without requiring the Fenton reaction (25).

Regarding RNS, these are also reactive species derived from nitric oxide and superoxide that are produced by nitric oxide synthase 2 and NAPDH oxidase, respectively.

### Salivary Antioxidant Capacity

**Figure 1:**
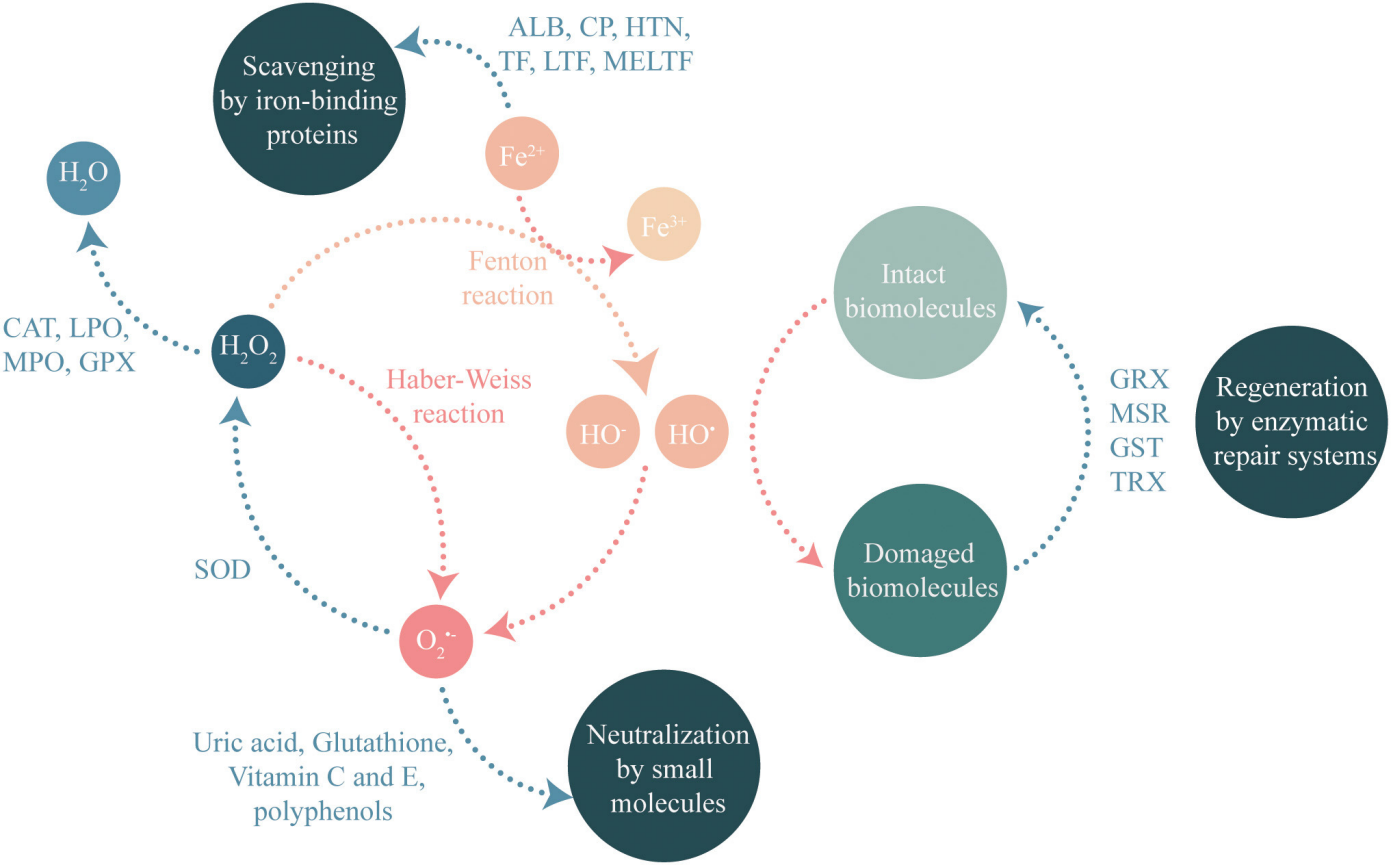
Molecular mechanisms controlling ROS concentration and participating to antioxidant capacity of saliva.

Saliva contains many molecules. Among them, many have antioxidant capacity that limits ROS generation. The salivary oral antioxidant capacity results from a combination of different molecular mechanisms (see Figure 1). For some reactions, the antioxidant power of these molecules requires enzymatic activities. Recent proteomic studies (26–29) have identified numerous antioxidant proteins or enzymes in saliva (see Table 1), thus providing insights into its antioxidant capacity.

**Table 1:** List of enzymes identified in proteomic studies that have an antioxidant activity.

**Process**	**Proteins**	**Genes**	**Accession numbers**	**Antioxidant functions**	**References**
ROS scavenging	Catalase	CAT	P04040	H_2_O_2_ detoxification	(32)
	Lactoperoxidase	LPO	P22079	H_2_O_2_ detoxification, hypothiocyanous ion synthesis	(36)
	Myeloperoxidase	MPO	P05164	H_2_O_2_ detoxification, hypothiocyanous ion synthesis	(36)
	Peroxiredoxins, isoforms 1 to 6	PRDX1 to PRDX6	Q06830, P32119, P30048, Q13162, P30044, P30041	Peroxides detoxification	(53)
	Superoxide dismutases, isoforms 1 to 3	SOD1, SOD2, SOD3	P00441, P04179, P08294	Superoxide radical dismutation	(30)
Redox maintenance and GSH-dependent enzymes	Glutaredoxins, isoforms 1, 3, and 5	GLRX, GLRX3, GLRX5	P35754, O76003, Q86SX6	Reduction of glutathionylated proteins	(53)
	Glutathione peroxidases, isoforms 1, 3, and 4	GPX1, GPX3, GPX4	P07203, P22352, P36969	Glutathione-dependent peroxides detoxification	(53)
	Glutathione reductase	GSR	P00390	Glutathione regeneration	(53)
	Glutathione transferases, isoforms from classes alpha, pi, mu, theta, and omega	GSTA1, GSTA4, GSTP1, GSTM1, GSTM2, GSTM4, GSTT1, GSTT2, GSTO1	P08263, O15217, P09211, P09488, P28161, Q03013, P30711, P0CG29, P78417	Xenobiotic detoxification, glutathione-dependent peroxides detoxification, ascorbate regeneration	(157)
	Methionine sulfoxide reductase A	MSRA	Q9UJ68	Reduction of methionine-sulfoxide in proteins	(58)
	Protein disulfide-isomerases	PDIA3, PDIA4, PDIA6, P4HB	P30101, P13667, Q15084, P07237	Disulfide bond formation	(53)
	Sulfhydryl oxidase	QSOX1	O00391	Disulfide bond formation	(53)
	Thioredoxin reductase	TXNRD1	Q16881	Dithiol-disulfide exchange reactions	(53)
	Thioredoxins, isoforms 1 and 2	TXN, TXN2	P10599, Q99757	Dithiol-disulfide exchange reactions	(53)
Metal binding	Albumin	ALB	P02768	Cd, Co, Cu, Fe, Hg, Ni, Zn binding	(158)
	Ceruloplasmin	CP	P00450	Fe, Cu binding	(159)
	Histatins, isoforms 1 and 3	HTN1, HTN3	P15515, P15516	Cu, Fe binding	(61)
	Lactotransferrin	LTF	P02788	Cu, Fe, Mn, Zn binding	(160)
	Melanotransferrin	MELTF	P08582	Fe binding	(161)
	Serotransferrin	TF	P02787	Fe binding	(162)

#### Elimination of ROS

To prevent the formation of the hydroxyl radical, specific enzymes were selected during evolution. Some enzymes remove superoxide anion thus preventing the Haber-Weiss reaction, while others detoxify hydrogen peroxide and thus prevent this same reaction but also the Fenton reaction. In addition, low-molecular weight molecules act directly by neutralizing ROS.

The main neutralizing enzymes are superoxide dismutases (SOD), which are metalloproteins that catalyze the dismutation of two ${\text{O}}_{2}^{\cdot -}$ molecules into H_2_O_2_ (30). These enzymes are the front line factors that directly remove superoxide anion (31). The formed hydrogen peroxide can then be eliminated by the catalase or peroxidase systems described in the following paragraph.

Catalase plays an important role because it allows NADPH-dependent dismutation of H_2_O_2_ into H_2_O and O_2_ (32). Historically, catalase activity measured in the saliva has been proposed to be mainly bacterial, with variability between healthy individuals and individuals with periodontitis (33). More recently, proteomic studies have shown that human catalase is also present in the saliva, thus indicating that salivary catalase activity has a dual human and bacterial origin (26). Catalase activity in the saliva is correlated with several diseases or habits. Salivary catalase activity is in increased in type I diabetics (34) but decreased in smokers (35). A second important family of enzymes involved in hydrogen peroxide detoxification are the peroxidases. Two peroxidases are present in the saliva: lactoperoxidase (also called salivary peroxidase) produced by the parotid and submandibular glands and myeloperoxidase contained in polymorphonuclear neutrophils (36). These enzymes, in addition to hydrogen peroxide reduction, also exhibit an antimicrobial potential through oxidation of the thiocyanate ion (36). The formed hypothiocyanite ion limits bacterial proliferation in the mouth by oxidizing thiol residues in essential microbial proteins (37) and can also inactivate human salivary detoxification proteins (38). Therefore, antioxidant systems appear essential in the maintenance of salivary redox balance.

In addition to enzymatic systems, low-molecular weight molecules also neutralize radical species. These molecules present diverse structures and functional groups (aromatic rings, hydroxyl groups). The main antioxidant molecule in human saliva is uric acid, which is responsible for nearly 70% of the antioxidant activity of saliva (39) with a concentration between 40 and 240 μM (39–42). The concentration of uric acid in the saliva correlates with its concentration in the plasma, suggesting that it comes from this fluid (43). Other small molecules are also involved in direct ROS scavenging including vitamin C (ascorbic acid) and vitamin E (tocopherols and tocotrienols) at cell membranes.

#### Maintenance of Salivary Redox Balance

Among the amino acid residues, cysteine and methionine are the most sensitive to oxidation. The oxidation of cysteine generates sulfenic acid that leads to the formation of a disulfide bridge after reaction with a second cysteine residue. In some cases, the oxidation of cysteine to sulfenic acid can lead to higher oxidation states, namely, sulfinic acid followed by sulfonic acid, which are more difficult to reverse. These oxidations cause the inactivation of thiol enzymes [e.g., cysteine proteases and salivary cystatins (44) or some salivary detoxification enzymes (38)], and can lead to the aggregation of salivary proteins (45). The reduction of thiols therefore enables to maintain the function of the salivary proteins containing thiols and additionally to absorb oxidation. Systems allowing thiol reduction (disulfide bridge or sulfenic acid) are therefore essential to maintain redox balance. At a concentration of ~600 μM (46), the main salivary thiol-type antioxidant is glutathione (tripeptide γ-L-glutamyl-L-cysteinyl-glycine). Glutathione can act directly on ROS (hydrogen peroxide and chlorinated oxidants), but its action on oxidized molecules can also be catalyzed by glutathione-dependent enzymes present in the saliva. Indeed, glutathione is a cofactor that allows electron transfer to reduce oxidized species. The oxidation of glutathione causes the formation of glutathione disulfide, and regeneration is carried out by glutathione reductase with NADPH as an electron donor. As a cofactor, glutathione is used by numerous salivary enzymes such as:

- glutathione peroxidase, which is involved in the glutathione-dependent decomposition of peroxides (47),
- glutathione transferases involved in the detoxification of xenobiotics and the elimination of lipid peroxidation products such as 4-hydroxy-2-nonenal (48),
- glutaredoxin, which ensures the reduction of glutathionylated proteins (49) and vitamin C (50). High levels of glutaredoxin have been detected in calf saliva (51). Several proteomic studies on human saliva have identified members of the glutaredoxin family (26, 27, 52).

Thioredoxin, the main enzyme involved in disulfide bridge reduction is found in human saliva (26–29). The thioredoxin system is made of thioredoxin reductase, which reduces thioredoxin, and NADPH, which is the final electron donor (53). Thioredoxin is multifunctional. In addition to its role as a regulator of protein thiol functions, thioredoxin can also directly neutralize certain ROS such as hydroxyl radical or singlet oxygen (54, 55). The levels of thioredoxin expression are increased in the salivary glands of patients with Sjögren's syndrome in response to oxidative stress induced by decreased salivary flow, thus protecting the salivary gland tissues (56). Thioredoxin has also been identified as a biomarker of appetite, with salivary levels modulated by food intake (57).

Oxidations can also occur at the level of methionine residues, generating methionine sulfoxides that can be reduced by methionine sulfoxide reductases (58). In cases of irreversible damage, proteolytic intracellular systems (cytosol proteasome and Lon protease in the mitochondria) eliminate the oxidized polypeptides (59). Many other enzymes involved in oxidation-reduction reactions have been identified from salivary proteomes (26–29). These include protein disulfide isomerases and sulfhydryl oxidase, which catalyze the formation of disulfide bridges, and peroxiredoxins, which catalyze the elimination of peroxides via the oxidation of cysteine residues.

Other salivary proteins may have antioxidant capacity without enzymatic activity mainly by preventing the Fenton reaction due to their metal-binding capacity (60). Salivary histatins have recently been shown to have antioxidant activity by inhibiting the formation of hydroxyl radicals generated during the Fenton reaction. This activity is likely explained by the chelation of the metal ions Fe^2+^ and Cu^2+^ (61). Histatins are low-molecular weight proteins that represent approximately 30% of the salivary proteins. With likely similar roles, we can also mention albumin (62), ceruloplasmin (63), transferrin (64), and lactoferrin (65).

#### Oxidative Damage Repair Systems

If the salivary redox state is out of balance leading to the ROS accumulation, biomolecule damages can occur. Depending on the nature of the damaged molecules, different repair systems are involved. In addition to the proteins discussed in the previous paragraph, lipids and DNA can also be affected. These systems, which involve enzymes that are not salivary but whose role is important for oral epithelial cell integrity, are briefly presented in the following lines. ROS can damage unsaturated lipids in the cell membranes by forming highly reactive radical products that are rapidly spreading through the formation of lipid peroxides (R-O-OH). In particular, vitamin E plays a major role in terminating the spread of damage and preserving membranes (66). Membrane-oxidized phospholipids can be specifically recognized and removed by lipolytic enzymes such as phospholipase A2 (67).

DNA is also sensitive to oxidation. Oral epithelial cell genomic alterations can be caused by oxidative stress generated by environmental pollution (68). Many DNA repair systems have been described (13). The diversity of the repair processes is related to the diversity of existing damage and the corresponding severity, as well as the need to preserve the integrity of the genetic information contained in the DNA. The main enzyme systems involved are basal excision repair (BER) for the repair of non-bulky lesions, nucleotide excision repair (NER) for the removal of bulky lesions affecting the three-dimensional structure of the DNA double helix and mismatch repair (MMR).

### Methodologies for Characterizing Salivary Redox Status

Salivary redox status is complex and involves many different types of molecules, making difficult its characterization. The most common biomarkers of oxidative stress reactions in the mouth are compounds resulting from lipid peroxidation, protein oxidation, and DNA oxidation and fragmentation products. Consequently, a first approach is to measure these oxidative stress biomarkers. As an example, malondialdehyde is a lipid oxidation product commonly measured as a salivary biomarker that results from the oxidation of polyunsaturated fatty acids (46). Malondialdehyde is titrated by its reaction with thiobarbituric acid, leading to the production of a compound absorbing at 535 nm (69).

A second approach to characterize salivary redox status is to measure the total antioxidant capacity and/or the different antioxidant molecules present in the saliva based on measuring the inhibition of free radical species formation. This method is based on the global measurement of the amount of reducing compounds (without distinction between chemical or enzymatic mechanisms). Total antioxidant capacity is defined as the sum of the antioxidant molecule activities in the saliva. Because these markers are involved in different biochemical pathways in human tissues, their concentrations are not necessarily correlated with each other (70). Thus, the use of a wide range of biomarkers can provide a better understanding of total antioxidant capacity (46).

Approaches to measure the total antioxidant capacity include the ferric reducing-antioxidant power (FRAP) test. This test quantifies the ability of saliva to reduce a colored Fe^3+^ complex by electron transfer to its corresponding Fe^2+^ complex. This method is based on measuring the formation of Fe^2+^- 2,4,6-tripyridyl-Striazine (TPTZ) or Fe^2+^-2,3-bis(2-pyridyl)-pyrazine (DPP) complexes, both absorbing at 593 nm, in the presence of saliva (71).

The amount of uric acid is another way to measure the total antioxidant capacity. Indeed, uric acid is both a preventive antioxidant (chelating activity) and a free radical scavenger. The measurement of uric acid is based on hydrogen peroxide production resulting from uric acid in the presence of the enzyme uricase. Hydrogen peroxide is quantified from the formation of the chromophore quinoneimine that absorbs at 500 nm (72). This chromophore is formed during the oxidation of p-hydroxybenzoate and 4-aminoantipyrine in the presence of peroxidase.

Antioxidant capacity measurements in Trolox equivalent (TEAC) evaluate the combined action of the different free radical scavengers present in saliva. This method is based on the scavenging of the radical cation 2,2′-azinobis-(3- ethylbenzothiazoline-6-sulfonate) (ABTS^∙+^). The formation of ABTS^∙−^ results from the activity of a peroxidase (metmyoglobin or horseradish peroxidase) in the presence of hydrogen peroxide and 2,2'-azobis-(2-amidinopropane) (LABA) and may be followed by measurement of the absorbance at 734 nm. The solution to be analyzed is added after the start of the reaction, and its ABTS^∙−^ scavenging activity is compared with that of a reference molecule, Trolox (73). Other methods for measuring the free radical scavenging ability of the saliva have also been described (74, 75).

Glutathione can exist in two forms: reduced (GSH) and oxidized (GSSG). Enzymes that use glutathione (cited in the precedent paragraph) modulate the ratio between these two forms (GSH/GSSG). The titration of these two forms allows the evaluation of their ratio in saliva. This method is based on the combined measurement of total and reduced glutathione. Reduced glutathione is quantified by reaction with 5,5′-dithio-bis (2-nitrobenzoic acid) (DTNB) forming the yellow 5′-thio-2-nitrobenzoic acid (TNB) derivative that absorbs at 412 nm. Measurement of the total amount of glutathione requires reduction of oxidized glutathione to GSH by a glutathione reductase in the presence of NADPH (76). The combination of these two methods therefore enables reduced glutathione and total glutathione quantification and thus one can deduce the amount of oxidized glutathione. Other methods based on electrochemistry can also be used to characterize reduced and total glutathione levels (77).

Importantly, the pre-analytical element of the experiment is crucial and should be taken into account when analyzing the results (78). Indeed, the variations in results between several studies may originate from pre-analytical differences. For instance, a common practice is to centrifuge the saliva before storing it at −20°C: however, a recent study demonstrated that some salivary enzyme activity may be lost during this step, leading to a reduction in inter-individual differences (9). Each protein eliminated by centrifugation that contains cysteine residues (i.e., with a reducing potential) can contribute to FRAP and TEAC indices. Each method has specific limitations: however, for each method the oxidation of the sample by air oxygen presents a major bias in addition to the loss of salivary protein activity.

## Relationship Between Salivary Antioxidant Capacity and Physiologic Status

### The Physiology of Saliva Secretion

Saliva is secreted by three major glands and numerous minors' glands, such as the von Ebner's glands. The major salivary secreting glands are the parotids, the submandibular and the sublingual. The origins of saliva are actually more complex because it is also composed of gingival fluid and the transudate of the oral and nasal mucosa (79). Saliva also contains bacteria and their metabolites, erythrocytes and cells resulting from the desquamation of the oral mucosa. Salivary gland secretions are neuronally and hormonally controlled (80). In non-pathological conditions, the salivary flow is between 0.75 and 1 L per day. Saliva is an aqueous fluid mainly composed of water that contains numerous organic and inorganic compounds, including salivary proteins that have numerous functions. Salivary pH is between 6.2 and 7.4 (81). As part of the saliva is the result of blood filtration, it can reflect the physiological status of the organism and is often called the “mirror of the body” (82). Indeed, the local vasculature of the salivary glands, the flow of gingival fluid and also intra-oral bleeding allow the passage of compounds from the systemic circulation into the saliva (83). Thus, the salivary concentration of compounds can reflect or can be directly correlated with systemic analytes (82). Moreover, saliva collection is non-invasive and does not required trained staff. Thus, saliva has been thought to be a good candidate to study the global redox status of the organism. However, saliva may not reflect the global physiological status of the organism, as it is a dynamic fluid and food diet and oral microbiota can impact on the salivary redox status.

Parotid saliva is the main source of antioxidants in the saliva. Parotid saliva contains much higher concentrations of various salivary molecules (uric acid) and enzymatic antioxidants (SOD and peroxidases) compared to the saliva secreted by the submandibular/sublingual glands (84). The parotid glands contribute to 20% of the total salivary flow, which is increased up to 60% under stimulated conditions. Thus, one can hypothesize that the higher antioxidant capacity of parotid saliva is aimed at combating deleterious foreign free radicals that may penetrate the body while eating. These observations also suggest that the oral cavity is less protected against ROS in resting conditions, which could increase the damage caused by smoking for instance (84).

Saliva could also have a role in the protection against lipid peroxidation. Indeed, parotid saliva has the ability to reduce peroxide of fatty acids (85). During the gastric phase, the pH of the gastric liquid enhances lipid peroxidation, which is catalyzed by the presence of food compounds such as Fe^2+^ or metmyoglobin in muscle tissues (86). In this condition, the presence of saliva allows partially inhibits lipid peroxidation (87).

This antioxidant role of saliva should also be considered for the formulation of salivary substitutes that could be used for patients presenting salivation troubles. For example, formulations using vegetable mucilage have shown interesting potential with regards to their viscoelastic and antioxidant properties. These substitutes clearly demonstrated their ability to scavenge ROS and chelate metallic ions (88).

### Relationship Between Physiological Status and the Antioxidant Capacity of Saliva

The antioxidant capacity of saliva decreases with age (89, 90). Moreover, structural changes are also observed with a decrease in salivary flow (91). These changes lead to increased oxidative stress in the oral cavity. Conversely, aerobic exercise decreases oxidative stress by increasing the concentration and the activity of salivary proteins that present antioxidant activity (92), such as salivary peroxidases (93).

Several studies have reported that obese people have a higher salivary total antioxidant capacity than normal weight individuals (10, 94, 95). Obese individuals reportedly have a higher salivary level of ferric-reducing antioxidant power, indicating a higher capacity of the saliva to chelate and inactivate metal ions (mainly Fe^2+^) involved in the formation of highly reactive ROS/RNS, including hydroxyl radical, and lipid peroxidation (95). In contrast, peroxidase activity in obese individuals has been described to be lower (94). At the same time, obese individuals show higher oxidative stress that is demonstrated by higher lipid peroxidation (95). Thus, the higher total antioxidant capacity in obese people is hypothesized to counterbalance their high oxidative stress (95).

### Relationship Between Oral Microbiota and Salivary Oxidative Stress

Microbiota and the total antioxidant capacity of saliva impact each other. Some microorganisms have the ability to limit the oxidative stress effect by producing antioxidant enzymes (96), while others produce ROS to limit the growth of other species and occupy the ecological niche: however, oxidative stress markers in the saliva are difficult to correlate with specific oral bacteria. No universal answer exists as to which specific bacterial species are associated with ROS production: the marker-species pairs can have negative correlations in some individuals while positive in others. Distinct intra-individual correlation patterns suggest that different bacterial consortia might be at the origin of oxidative stress induction (70). This hypothesis can be explained by the fact that cells belonging to the same bacterial species are distributed in different compartments of the oral cavity with different physiological optimums. While some cells actively metabolize nutrients, others wait for optimal growth conditions in a dormant stage (70). The microbiome composition can also change during the day (97). Thus, studies on the relationship between oxidative markers and bacteria composition require the collection of multiple samples per individual at different times during the day. The consumption of fermented foods, such as cheese, can lead to a temporary modification of the composition of the microbiota and is associated with a temporary increase in oxidative stress (98). Other studies have demonstrated a positive correlation between the intake of simple carbohydrates and the salivary total antioxidant capacity (99). This observation could be due to increased uric acid in the blood (100, 101). This correlation could also result from an increase in bacteria involved in dental caries formation, leading to an increase in oxidative stress, which could be controlled by increased levels of antioxidant species in the saliva (99).

## Links Between Salivary Redox, Diet and Food Perception

### Relationship Between the Total Antioxidant Capacity of Saliva and Diet

Numerous analyses have revealed links between salivary oxidative stress and the pathologies of the oral cavity, including cancers (27, 102), odontogenic cyst (103), lichen planus (104), Sjögren's syndrome (56), and chronic periodontitis (105, 106), leading to the exploration of whether food containing antioxidant compounds or diet supplementation can modulate oral antioxidant capacity (see Table 2). Table 2 presents a list of food and food molecules for which an antioxidant effect has been reported. Most studies have analyzed vitamin supplementation and the effect of food rich in polyphenols.

**Table 2:** List of food and food molecules that have an antioxidant effect.

**Type**	**Molecules or food**	**Effects**	**References**
Food	Apple	Inhibit nitrosation/nitration but also promote NO bioavailability at the gastric level	(163)
Food	Broccoli and coffee	Increase in the secretion of glutathione transferase	(131)
Food	Bryndza cheese	Decrease in salivary TAC	(98)
Food	Coffe and Chlorogenic acid	Decrease in RNS	(113)
Food	Cranberry	No effect on salivary total antioxidant status	(129)
Food	Green Tea	Increase in the salivary TAC in chemical laboratory workers	(125)
Food	Green Tea	Increase in the salivary TAC in elderlies	(126)
Food	Green Tea	Increase in the salivary TAC in smokers	(127)
Food	Green Tea	No effect on free radical scavenger activity after exercise training	(164)
Food	Polyphenols in red wine	Offset in the oxidative impact of ethanol	(132)
Food	Sea buckthorn oil extract	No effect on oxidative markers and salivary flow	(165)
Food	Tannins	Increase in the secretion of histatines	(136)
Food	Wine	Presence of polyphenols in wine preclude the oxidative effect of ethanol	(132)
Molecules	Astaxanthine	Decrease in the level of salivary oxidative stress via ROS scavenging	(141)
Molecules	Caffeine	Inhibit human salivary aldehyde dehydrogenase	(130)
Molecules	Flavan-3-ol	Galloyl-containing polyphenols promote iron oxidation at a significantly faster rate than analogous catechol-containing compounds, and iron oxidation rate also correlates with polyphenol inhibition of DNA damage for polyphenol compounds	(116)
Molecules	Isoflavone supplementation in addition to combined exercise	Decrease in nitrite and thiobarbituric acid reactive substances and no effect on total antioxidant capacity or total protein	(166)
Molecules	Nitrate	Increase in nitrite and uric acid concentration in saliva	(142)
Molecules	Omega-3-fatty acids	No effect on the SOD level in saliva	(110)
Molecules	Quercetin	Enhanced the formation nitric oxide	(113)
Molecules	Quercetin	Reduction of nitrous acid to nitric oxide	(114)
Molecules	Resveratrol	Protect salivary glands against the negative effects of irradiation	(128)
Molecules	ß-carotene	No effect on salivary TAC and C- reactive proteins	(108)
Molecules	ß-carotene	No effect on Non-Urate Total Antioxidant Capacity in elderlies	(109)
Molecules	Vitamin C	Increase in the antioxidant status; Decrease in carbonyl stress	(107)
Molecules	Vitamin C	No effect on TAC	(167)
Molecules	Vitamin C	No effect on salivary TAC and C- reactive proteins	(108)
Molecules	Vitamin C	No effect on Non-Urate Total Antioxidant Capacity in elderlies	(109)
Molecules	Vitamin C	No effect on the SOD activity in saliva	(168)
Molecules	Vitamin E	No effect on salivary TAC and C- reactive proteins	(108)
Molecules	Vitamin E	No effect on Non-Urate Total Antioxidant Capacity in elderlies	(109)
Molecules	Vitamin E	No effect of SOD activity in saliva	(169)

Indeed, as vitamins are involved in the regulation of the oxidative stress, different studies have investigated the impact of vitamin supplementation with contradictory results. Vitamin C, also known as ascorbic acid, is an antioxidant naturally present in saliva at 2.5 μg/mL. Vitamin C is involved in a variety of hydroxylation reactions. Kamodyova et al. observed a positive effect of vitamin C supplementation (250 mg) on the salivary antioxidant status using two tests, the TAC (TEAC test; average increase of 1%; *p* < 0.01) and on the ability of saliva to chelate and inactivate metal ions (FRAP test; average increase of 107%; *p* < 0.01). At the same time, vitamin C supplementation had a negative impact on carbonyl stress (markers of advanced glycation end products; average decrease of 64%; et *p* < 0.001) (107). Other studies reported that the level of vitamin consumption does not modify antioxidant markers in the elderly (108, 109). The effect of omega-3 fatty acids has also been investigated and revealed no effect on the level of SOD enzymes (110).

Polyphenols, known for their antioxidant properties, are ubiquitous in plant and plant-based foods, and more particularly in tea and berries. Indeed, the antioxidant activity of plants depends on their concentration of polyphenols, and tea and berries contain high polyphenol levels (111). Two mechanisms could be involved in the antioxidant capacity of polyphenols: (i) scavenging of ROS (112) and RNS (113, 114) and ion chelation (115). The chelation of Fe^2+^ ions by polyphenols increases their oxidation to Fe^3+^ ions in the presence of oxygen. This effect depends on the polyphenol structure and is increased when Fe^2+^ ions are bound to a galloyl group (116). The chelation of Fe^2+^ ions with their oxidation to Fe^3+^ ions decreases the quantity of Fe^2+^ that could participate in the Fenton reaction that is at the origin of the production of hydroxyl radicals (116). Thus, red wine tannins inhibit lipid peroxidation of muscle tissues during the gastric phase, whereas peroxidation is only partially inhibited in the presence of saliva alone (87): however, the interaction between polyphenols, especially those having galloyl groups, and salivary proteins, in particular proline rich-proteins (117) or histatins (118), could decrease the antioxidant activity of polyphenols by competing with their binding to Fe^2+^ (109, 119). The effect of polyphenol binding to salivary proteins is not so simple. The interactions of salivary proteins, including mucin, with polyphenols can increase the antioxidant activity of lipophilic polyphenols (120–122) by increasing their solubility. Additionally, these interactions could allow some polyphenols to remain in the oral cavity several hours after consumption (122). This mechanism could be particularly important if the proteins composing the mucosal pellicle are involved, as they are anchored at the surface of the oral epithelial cells (123) and are not swallowed. Monomers of flavan-3-ol also have the ability to bind to lipids present in food and membrane cells and to protect them from oxidation (124).

Many studies have examined the impact of polyphenol rich foods on the antioxidant capacity of saliva. For instance, green tea has been particularly studied because it contains high levels of the oligomers of flavan-3-ol. Green tea reportedly increases the total antioxidant capacity of saliva in several specific populations: chemical laboratory workers (FRAP method; 22% increase; *p* = 0.016) (125), the elderly (TEAC method; 42% increase; *p* < 0.001) (126) and smokers (FRAP method; 43% increase; *p* < 0.001) (127). Green tea intake appears to partially compensate for differences in TAC between smokers and non-smokers (127). In rats, resveratrol intake can protect salivary glands and salivary proteins, including SOD, against the negative effects of irradiation (an important source of oxidative stress) (128). Conversely, another study reported that intake of cranberry juice, which is rich in polyphenols, does not impact systemic or salivary TAC (129).

In addition to polyphenols, oxidant molecules can be present in food, such as caffeine in coffee. Caffeine inhibits salivary aldehyde dehydrogenase, which has an antioxidant function and participates in the detoxification of toxic aldehydes within the oral cavity (130). In response, the secretions of salivary aldehyde dehydrogenase and glutathione transferase are increased (131). This mechanism, which leads to increased secretion of glutathione transferase, has also been observed for Brassicaceae intake including broccoli (131). Red wine is another example of food containing both pro- and antioxidant compounds. The presence of polyphenols in red wine may be able to counteract, at least in part, the pro-oxidant activity of ethanol (132). Ethanol is metabolized by alcohol dehydrogenase (ADH) and cytochrome P450 into acetaldehyde (ethanal) and NADH or acetaldehyde and ROS. Acetaldehyde can inhibit the activity of salivary peroxidases (lactoperoxidase and myeloperoxidase) (133). As a result, an increase in the activity of salivary peroxidases correlated to a decreased salivary flow in alcoholic patients (134). Whether this increase in salivary peroxidase activity aims at compensate its inhibition by acetaldehyde or if it originates from an influx of leukocytes into damaged oral mucosa remains unknown (134).

Moreover, the effect of polyphenols could be indirect. In mammals, a tannin rich diet induces increased secretion of tannin-binding salivary proteins in the saliva of herbivores (135, 136). Tannin-binding salivary proteins are mainly composed of two salivary protein families: proline-rich proteins and histatins (137). These proteins have demonstrated a high affinity for tannins (118, 138) due to their structure (117, 139) and play a role in the protection of the oral mucosa (140). Histatins have recently been shown to have antioxidant activity, as they are able to decrease the production of hydroxyl radical likely through the binding and scavenging of Fe^2+^ ions or through Fe^2+^ oxidation to Fe^3+^ (61). Thus, an increased histatin concentration in the saliva could increase the antioxidant capacity of the oral cavity.

Carotenoids are another class of molecules present in plants and animals that have antioxidant properties that could impact salivary antioxidant capacity. For instance, astaxanthin, a carotenoid with strong antioxidant properties, is found in fishes such as sea bream and salmon as well as crustaceans such as crab and shrimp. Diet supplementation with astaxanthin induced a decrease in oxidative stress at the salivary level (measurement of the lipid peroxidation marker hexanoyl-lysine; 10% decrease; *p* = 0.03). This molecule appears to scavenge ROS (141). A rich nitrate (${\text{NO}}_{3}^{-}$) diet can also affect the antioxidant capacity of saliva. Nitrate is ingested as a food component. The nitrate ingested is absorbed from the intestine into the bloodstream and is then secreted into the saliva. In the saliva, nitrate is reduced to nitrite (${\text{NO}}_{2}^{-}$) by certain bacteria, and the nitrite formed is reduced to nitric oxide (NO). Then, nitric oxide can react with molecular oxygen to produce NO_2_ and N_2_O_3_.

$$\begin{array}{l} {\text{2NO+O}}_{\text{2}}\to {\text{2NO}}_{\text{2}}\\ {\text{NO}}_{\text{2}}\text{+NO}\to {\text{N}}_{\text{2}}{\text{O}}_{\text{3}} \end{array}$$

NO can also react with $O_{2}^{-}$ that is produced by bacteria forming ONOO^−^.

$${\text{NO+O}}_{\text{2}}^{-}\to {\text{ONOO}}^{-}$$

NO_2_ and ONOO^−^ are oxidizing and nitrating agents and thus reactive species. In acidic conditions, nitrite can be protonated and form nitrous acid, which decomposes to various nitrogen oxides. The RNS generated contribute to oxidative stress in the oral cavity (113, 114): however, polyphenols, such as quercetin, are able to reduce nitrous acid to nitric oxide (113). A study on the impact of nitrate supplementation after physical exercise for 5 days reported an increase of nitrite and uric acid in the saliva. Moreover, lipid peroxidation and SOD activity were decreased after 30 min in supplemented subjects. The decreased lipid peroxidation can be explained by the increased NO resulting from the nitrite, which acts as an inhibitor of lipid peroxidation by scavenging peroxyl radicals (142).

Last, eating, especially mastication, plays an indirect role in salivary antioxidant capacity. The parotid glands of experimental animals fed with a liquid diet reportedly show atrophy (143). Parotid gland atrophy is due to decreased parasympathetic nerve stimulation, which is involved in the proliferation of the parotid glands (144). Thus, a lack of sufficient masticatory force might lead to a reduced masticatory-parotid reflex and consequently lead to atrophy of the salivary glands. Parotid gland atrophy leads to decreased parotid salivary flow and thus to salivary antioxidant capacity. Indeed, enteral nutrition feeding alters salivary antioxidant capacity by decreasing the concentration of uric acid and the total protein content in the saliva (145). Another study demonstrated that children with eating difficulties resulting from enteral or parenteral nutrition in neonatal periods have a lower antioxidant status (146): however, supplementation of a liquid diet with L-carnitine in rat prevented atrophy of the parotid glands. L-carnitine has been hypothesized to protect the mitochondria and endoplasmic reticulum from oxidative stress that results from decreased cellular energy production (147). Being a potential scavenger of ROS, L-carnitine is an antioxidant that prevents the impairment of fatty acid oxidation in the mitochondria.

### Relationship Between Food Flavor Perception and Salivary Antioxidant Capacity

During the last decade, several studies have considered the role of salivary antioxidant capacity in flavor perception. Indeed, flavor molecules are subject to oral and nasal metabolization as a function of their structure. These reactions affect both the quality and the quantity of flavor compounds available to activate the chemosensory receptors. Different results have suggested that these reactions may be modulated by salivary antioxidant capacity (8–10). A recent article showed a highly significant positive correlation between salivary TAC and taste disorder in 120 patients compared to normal subjects. Increased TAC was associated with increased catalase and SOD activities (148).

The interest to gain a deeper understanding of these mechanisms has grown with the recent demonstration that nasal and oral metabolic activity of aroma compounds impact aroma perception (12, 149). Thus, several studies have investigated the impact of salivary antioxidant capacity on the perception and release of aroma compounds in addition to the role in fatty acid perception.

Several studies on both normal subjects (9) and specific populations including obese (10), elderly (11) or the elderly suffering from hyposalivation (8) have observed a negative correlation between salivary TAC and aroma release in the presence of the subjects' saliva. This effect depended on the structure of the aroma compounds and was related to the chemical reactivity of the molecules. Whereas, some ketones and aldehydes were strongly affected, the studied alcohols were not metabolized (9). Indeed, Muñoz et al. suggested that the decreased release of specific aroma compounds results from their metabolization by salivary xenobiotic-metabolizing enzymes. Xenobiotic-metabolizing enzymes are involved in the clearance and deactivation of xenobiotics, including aroma compounds. This effect is achieved through two enzymatic steps involving different classes of xenobiotic-metabolizing enzymes. Phase I enzymes catalyze the biotransformation of compounds with reactive chemical functions, including carbonyl groups, forming metabolites carrying less reactive functional groups (OH, NH_2_, COOH). Phase II enzymes, including glutathione transferases (GST), can promote the biotransformation of compounds naturally functionalized or phase I metabolites by catalyzing their conjugation with hydrophilic moieties (e.g., glutathione). Phase II leads to hydrophilic conjugates of the initial molecules, including glutathione conjugates of some flavor compounds such as cinnamaldehyde (150). Some of these enzymes are NAD(P)H dependent (9). The TAC provides information on the redox status and indirectly on the equilibrium of the balance of [NAD(P)^+^]/[NAD(P)H] and thus on the level of activity of these enzymes. Indeed, addition of NADH to the saliva significantly increased the enzymatic degradation of octanal into octanol (9). This metabolic activity has also been reported in the presence of oral epithelial cells (151). Coffee or broccoli intake has been reported to increase the secretion of xenobiotic-metabolizing enzymes, including GST, resulting in a higher metabolic activity at the salivary level (131). This mechanism is believed to impact perception by modulating the quality and quantity of flavor compounds (6).

Beside this metabolic activity, lipid oxidation in the oral cavity leads to the formation of volatile aldehydes and ketones that can have a metallic aroma note and as a result participate in the perception of a metallic taste. In fact, metallic taste is more a flavor as it probably results from the cerebral integration of different senses leading to a multimodal perception. Lipid oxidation, especially of polyunsaturated fatty acids, is highly increased in the presence of Fe^2+^ ions and to a lesser extent by Fe^3+^ (152). This oxidation is precluded in the presence of molecules that are able to chelate Fe^2+^ ions, and only partially reduced in the presence of vitamin C, an antioxidant that is able to scavenge free radicals (152). Indeed, vitamin C can also reduce Fe^3+^ to Fe^2+^, which will participate to the formation of ROS via auto-oxidation or the Fenton reaction (153, 154): however, no correlation between the salivary TAC, metallic taste perception and the production of aldehydes and ketones resulting from lipid oxidation has been observed (152).

Concerning fat perception, Poette et al. have reported a positive correlation between the detection threshold of non-esterified fatty acids without nose-clip and the antioxidant status of saliva, whereas there is no correlation with nose clip (155). These results suggest that the metabolization or the oxidation of non-esterified fatty acids leads to the release of aroma compounds involved in fat perception. Thus, as for metallic taste, fat perception appears to be a multimodal perception involving both the perception of aroma compound release in the oral cavity, trigeminal perception (mechanoreceptors involved in the perception of texture) and potentially taste chemoreceptors dedicated to the detection of free fatty acids such as the CD36 receptors located in the taste buds (156).

## Conclusion

Salivary antioxidant capacity involves numerous molecular species that can counterbalance their respective activity. Moreover, feeding and associated pathologies (obesity, alcoholism, etc.) can increase salivary oxidative stress, while the consumption of antioxidants can increase salivary antioxidant status: however, the direct association of foods with salivary antioxidant capacity remains fragile and contradictory results on the effect of antioxidant intake highlight the necessity for future studies in this area, which should consider more than one antioxidant marker to account for the different biochemical mechanisms. Such investigations should provide a deeper understanding of the respective contribution of the different mechanisms involved. Another promising area of research concerns the relationship between salivary antioxidant capacity and the perception of flavor. Indeed, flavor corresponds more to the perception of the sum of flavor molecules and metabolites formed in mouth than food flavor molecules alone. Highly toxic flavor compounds are hypothesized to be quickly eliminated by xenobiotic-metabolizing enzymes at the salivary level before they reach the chemosensory receptors. Thus, the metabolites formed by the detoxification pathway can themselves provide information on the potential toxicity of food. Several markers of salivary antioxidant status should be considered to preserve the interindividual variability of saliva.

## Author Contributions

MS, FN, and FC drafted the manuscript with input from all authors. GF, MS, FN, and FC revised the manuscript. All authors have approved the submitted version.

## Conflict of Interest

The authors declare that the research was conducted in the absence of any commercial or financial relationships that could be construed as a potential conflict of interest.
